# Amazon tropical fishes of commercial interest show human-cell contamination but no SARS-CoV-2 in a real-life scenario

**DOI:** 10.1371/journal.pone.0306985

**Published:** 2024-07-15

**Authors:** Carolina Sousa de Sá Leitão, Carlos Henrique dos Anjos dos Santos, Jefferson Valente, Bernardo Maia, Rogério Santos Pereira, Larissa Matos Batista, Felipe Guedes Amorim, Luciana Mara Fé-Gonçalves, Marcus Lacerda, Fernando Almeida-Val, Adalberto Luis Val

**Affiliations:** 1 Laboratório de Ecofisiologia e Evolução Molecular, Instituto Nacional de Pesquisas da Amazônia, Laboratório de Ecofisiologia e Evolução Molecular, Manaus, Amazonas (AM), Brasil; 2 Unidade Acadêmica de Serra Talhada, Universidade Federal Rural de Pernambuco, Serra Talhada, Pernambuco (PE), Brasil; 3 Universidade do Estado do Amazonas, Manaus, AM, Brasil; 4 Fundação de Vigilância em Saúde Dra. Rosemary Costa Pinto, Manaus, AM, Brasil; 5 Fundação de Medicina Tropical Dr. Heitor Vieira Dourado, Manaus, AM, Brasil; 6 Instituto Leonidas e Maria Deane, FIOCRUZ-AM, Manaus, AM, Brasil; University of Alcala Faculty of Medicine and Health Sciences: Universidad de Alcala Facultad de Medicina y Ciencias de la Salud, SPAIN

## Abstract

**Background:**

Amazonas was one of the most impacted Brazilian states by the COVID-19 pandemic. Mortality rates were high, and the health systems collapsed. It is important to identify possible intermediate reservoirs to avoid animal-to-human contamination. Several tropical fish are of commercial interest and are sold in large open-air markets in the region, representing a large economic and dietary importance.

**Objectives:**

This study aimed to verify if fish species of commercial importance, aerosols, and fish wastewater in local open-air markets, at a major capital city in the western Brazilian Amazon, are contaminated by SARS-CoV-2.

**Methods:**

488 fish, 50 aerosol, and 45 wastewater samples were analyzed for the presence of SARS-CoV-2. The samples were subjected to extraction using the BIOGENE Viral DNA/RNA Extraction kit, and the molecular diagnosis was tested for SARS-CoV-2 using the Bio-Manguinhos SARS-CoV-2 (EDx) Molecular Kit.

**Results:**

It was not possible to detect the virus (Ct≤40, for Gene E) in these samples, however, in 181 samples of fish it was possible to detect the human RP gene (Ct≤35, for the RP Gene), indicating human contact. There was a high number of COVID-19 diagnoses in all city districts in which the samples were collected, showing that SARS-CoV-2 was circulating.

**Conclusion:**

This study indicates that fish of local commercial importance do not carry SARS-CoV-2 viral particles, despite circulation of SARS-CoV-2, and are not an important source of animal-to-human contamination. Despite these results, the human RP gene was found detectable in fish, air, and fish wastewater, showing that such places may carry human pathogens.

## Introduction

Since the first reports of cases of COVID-19 [1], and the identification of the SARS-CoV-2 as its causative agent [2], there have been more than 700 million cases and almost 7 million deaths worldwide [3]. Completing three years, the pandemic has challenged the public, sanitary, and health systems in an unprecedent manner and disruptions are still evident [2]. The origin of the virus remains unclear, but current data show that the virus is the result of multiple zoonotic transmissions due to susceptible live wildlife marketing [4]. Nonetheless, evidence also suggests that it is unlikely that the SARS-CoV-2 circulated in humans before late October 2019 [5].

The main spread of the SARS-CoV-2 virus is human-to-human, although, animal-to-animal and spillback through human-to-animal transmission have been documented [6]. A wide variety of animal infections has also been demonstrated including those occurring in vertebrate animals as in the pet, captive, wild, and farmed animal categories [7]. This is of concern once the virus has demonstrated a “generalist” nature with little need to adapt to another animal in human-to-animal spillovers [8].

The epidemiological dynamics and ecology of disease in humans and animals should be monitored closely. The establishment of surveillance tools in this manner could enhance overall public health interventions while also preventing pathogen establishment in novel animal hosts. Sources of infection, such as fresh and open-air food sold in markets, or in frozen food sources in cold chain transportation, are thought to be a threat to public health, especially in low- and middle-income countries since regulations on dead and alive wildlife marketing are not closely followed. For instance, in China, several sporadic outbreaks of COVID-19 in 2020 were linked to food in cold chain sold at trade markets, including salmon meat [9], with the detection of SARS-CoV-2 RNA on the surface of frozen meat for as long as 20 days [10]. This is alarming in places where a great part of the population is economically and nutritionally dependent on these activities, as in the Amazon region.

Given the epidemiology of COVID-19 in Brazil and, particularly, Brazilian Amazonas state, it is paramount to identify possible transmission reservoirs of SARS-CoV-2. Among the possibilities of intermediate hosts, a main question arises in this context: would aquatic animals used as human food be part of this cycle in the Amazon region? In this study, we aimed to: (i) verify the exposure of the marketed fish specimens to human contact through quantification of the human gene (RP) in the fish samples; (ii) identify if the marketed fish in local open-air markets presented SARS-CoV-2 deposited in their skin tissue and quantify the viral RNA; (iii) collect aerosol samples (atmospheric air) from the open-air fish markets to verify the presence of SARS-CoV-2 in these localities; (iv) analyze the residual presence of SARS-CoV-2 in fish wastewater; and (v) verify the circulation of SARS-CoV-2 in neighborhoods surrounding the fish markets at the respective time samples from fish, air and wastewater were collected.

## Methods

### Study design

This study was designed to investigate whether fish used for human consumption and sold at open-air fish markets in Manaus, the capital of the Amazonas state, located in the Western Brazilian Amazon, could be a possible source of infection of SARS-CoV-2 to humans. Therefore, this study encompassed a transversal analysis of fish specimens, air, and wastewater collected from several open-air fish markets in Manaus and an epidemiological approach to estimate viral circulation in the surrounding neighborhoods to these markets.

### Sample collection from open-air fish markets

Specimens from fish popularly commercialized in open-air markets, known as aracu (*Anostomoides laticeps*), curimatã (*Prochilodus nigricans)*, jaraqui (*Semaprochilodus insignis*), matrinxã (*Brycon amazonicus*), pacu (*Metynnis lippincottianus*), pirarucu (*Arapaima gigas*), sardinha (*Triportheus elongatus*) and tambaqui (*Colossoma macropomum*) were collected and used in this study. These specimens were collected from the open-air markets distributed in the city of Manaus presented in Fig 2, in the capital of the Amazonas state. Swabs from the skin of the species mentioned above were collected according to the World Health Organization protocol for surface sampling [11]. Briefly, swabs from fresh fish were moistened in sterile water and the material rotated over the fish skin. Subsequently, the swabs were stored in individual tubes containing a 5 mL transport solution for preservation and virus inactivation. The material was kept under refrigeration (-80°C) until the moment of laboratory analysis.

Air for aerosol collections was carried out using air samplers containing 5 mL of solution for transporting and preserving the virus. The collection was performed using a vacuum pump, with a speed of 17 L.min^-1^, in bottles containing 20 mL of 0.9% saline solution, for 1 hour, equivalent to 1.02 m^3^ of air. The air collector generated an air flow towards the transport solution with a flow rate of six liters per minute. The entire procedure was timed to standardize the collections. The processing of aerosol samples followed the same procedures adopted for fish samples.

The fishing trade chain involves the harvesting of fish from freshwater waterbodies within the local riverine environment. For such, fishermen collect and stock fish specimens in boats with ice from the moment of the catch to the market exposure to the population. In this process, the fish are exposed to air, which may contain SARS-CoV-2 aerosol particles from infected individuals. Before exposing these fish to the public, specimens are washed with water. Aiming to verify if SARS-CoV-2 was present in the fish washing water, specimens were collected and cleaned three times with running water. Afterward, the water was kept under refrigeration (-80°C) until the moment of laboratory analysis.

### Molecular diagnosis of SARS-CoV-2

The samples (fish skin, and market air and wastewater) were subjected to extraction using the BIOGENE Viral DNA/RNA Extraction kit, according to the manufacturer’s recommendations. For molecular diagnosis, samples were tested for SARS-CoV-2 using the Bio-Manguinhos SARS-CoV-2 (EDx) Molecular Kit. For molecular diagnosis, samples were considered positive if both, Gene E (Sars-Cov2) and Gene RP (Human) presented a threshold of Ct ≤ 40, for Gene E, and Ct ≤ 35 for Gene RP [12].

### SARS-CoV-2 viral transmission in fish markets

To investigate SARS-CoV-2 viral transmission in the neighborhoods surrounding these fish markets, we decided to use the viral circulation proxy, an epidemiological evaluation technique that consists of identifying positive cases in individuals residing in the region, to assess the local viral circulation. For this, we collected data from an official public source of COVID-19 laboratory-confirmed cases. The Fundação de Vigilância em Saúde Rosemary Costa Pinto (FVS-RCP) is responsible for coordinating COVID-19 surveillance systems in the Amazonas state [13, 14]. SARS-CoV-2 diagnosis and probable infection site are examples of data that were collected and used here to show COVID-19 cases according to fish market neighborhoods at the time of fish specimens’ collection. The evaluation was carried out considering the period of collection of fish samples (until May 2021).

### Statistical analysis

Data presentation is mainly descriptive. When necessary, data was summarized, and distribution was tested by the Shapiro-Wilk test. Mean and standard deviation, or medium and interquartile ranges, were used to represent normal and abnormal distributed data, respectively. The Spearman Chi-square test was used to compare the distribution of variables within groups. A total number of cases are also presented when appropriate. The images were created for this study using the QGIS software, version 3.30.2.

### Ethical considerations

The entire study was conducted following the ethical regulations for research with animals in Brazil, based on the guidelines of international practices with animals. Approval for animal use in this study from the local institutional review board for animal use was not necessary since the specimens were no longer living at the time of the experiments. Data for SARS-CoV-2 circulation in neighborhoods surrounding the fish markets were obtained from a public resource and no approval was needed. No human samples were used in this study. All research staff were trained and harmonized before study procedures were performed.

## Results

**Fig 1:**
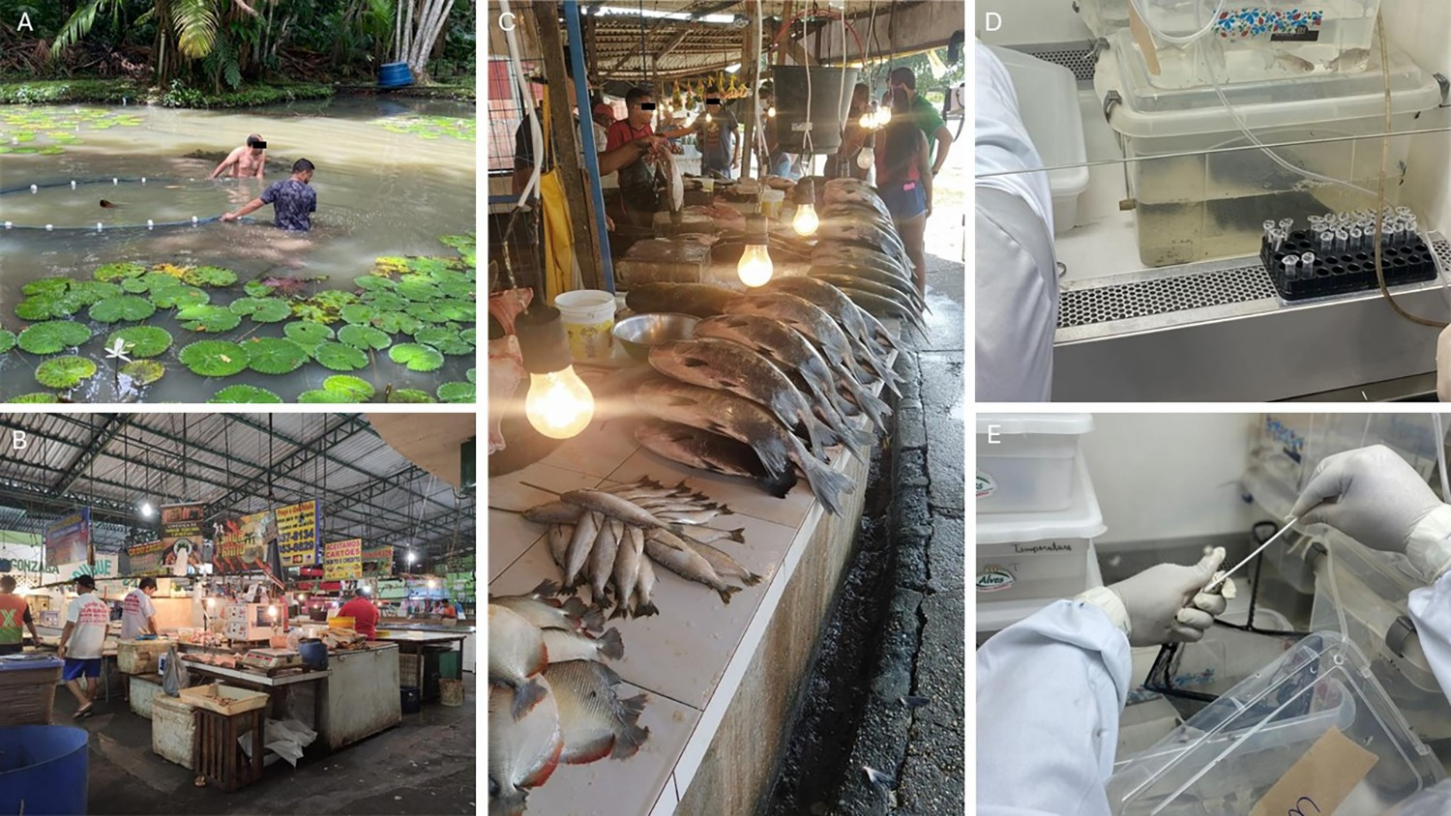
A—fishing activities; B—fish market; C—fish exposed to ambient air in the fish market; D—laboratory procedures and sample collection; E—fish swabbing procedures.

**Fig 2:**
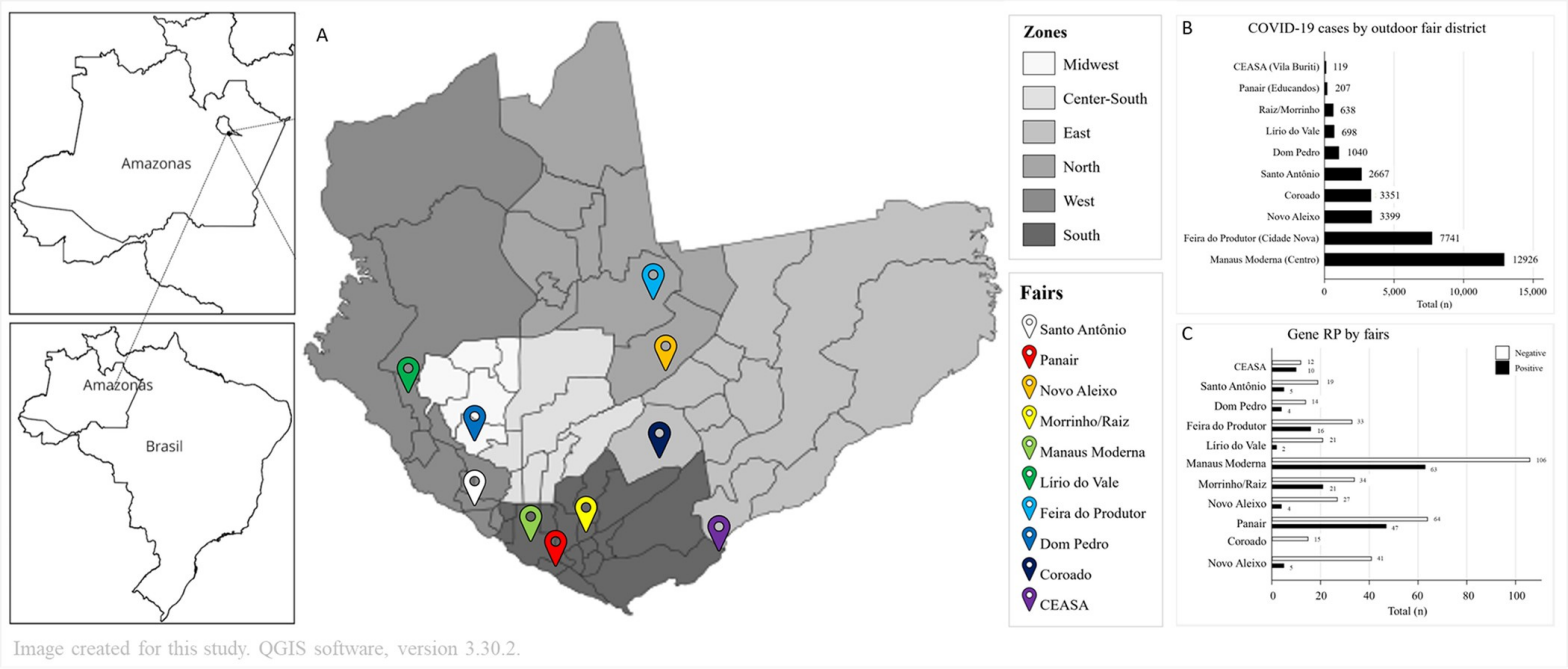
(A) Geographical distribution of open-air fish markets in the city of Manaus, according to city zones, with most of them concentrating in the riverine portion of the city; (B) COVID-19 cases in the city districts where the open-air fish markets are distributed (May 2021), the same time that the fish specimen collections were performed. Of relevance, most of the fish markets are in regions where a higher number of COVID-19 cases were reported, showing a high circulation of the virus at that time; (C) the presence of the human RP gene, which is classified as positive and negative according to the open-air market and shows that there was a predominance of positivity to human exposure within the collected specimens.

A total of 488 specimens of fish of commercial interest for the local population were collected. Steps from fishing to laboratory procedures are depicted in Fig 1. These included 33 specimens of aracu, 45 of curimatã, 86 of jaraqui, 27 of matrinxã, 92 of pacu, 43 of pirarucu, 91 of sardine, and 71 of tambaqui. These were carried out in a total of ten fish open-air markets located in the following neighborhoods of Manaus city (Santo Antônio, Panair, Novo Aleixo, Morrinho/Raiz, Manaus Moderna, Lírio do Vale, Feira do Produtor, Dom Pedro, Coroado and CEASA, which are shown in Fig 2A). The total number of cases in each neighborhood is shown in Fig 2B and the number of fish specimens presenting the human RP gene is shown in Fig 2C.

A total of 50 air samples were collected and the aerosol was analyzed for SARS-CoV-2. The virus was not detected in free circulation carried by aerosols (atmospheric air) in these places. Also, a total of 45 samples of wastewater were collected (from the washing of fish and the resale spaces at the marketplaces) and showed no amplification signal corresponding to the contamination of SARS-CoV-2. However, 181 samples of fish showed amplification of the human RP Gene (Fig 2). Considering the outdoor fairs, the RP gene was identified in 181 samples, in greater quantity in the Manaus Moderna fair (35.5%, 63/177), followed by the Panair fair (26.5%, 47/177). Despite a lower percentage, the human RP gene was also present in aerosol and wastewater samples from the open-air fish markets.

## Discussion

In this study, we collected 488 samples of food fish and tested the hypothesis that this animal’s skin surface could be a possible intermediate reservoir for the virus transmission. However, no SARS-CoV-2 amplification was observed on the fish’s surface. We also collected air and fish wastewater from open-air fish markets and observed if they carried the SARS-CoV-2 virus, but this also turned negative. We also studied the presence of the human RP gene in such specimens and materials to evidence the feasibility that human contact could be a possible source of the spreading of genetic material, including those causing diseases, which we found to be positive. Additionally, we verified the overlap of the probable infection place of COVID-19-positive individuals and the open-air markets to view, as a proxy, the viral shedding in these localities. This last analysis, in conjunction with the other presented data in this study, showed that there was viral shedding in surrounding neighborhoods, with infections seeming to be exclusively caused by human-to-human interactions.

Surfaces in public areas that are highly exposed to human crowding, as in fish markets, or that are frequently touched by the hands (e.g., fresh food) are thought to frequently harbor positive for SARS-CoV-2 RNA. This has been shown true in ATM machines [15]. Furthermore, the SARS-CoV-2 viral RNA has been detected on environmental surfaces in several places, such as playgrounds [16], supermarkets [17], cruise ship surfaces [18], public transport vehicles [19], tourist recreational facilities [20], and workplaces [21], all of which are common in tourist cities such as Manaus. The atmospheric air of the markets, the surfaces of the fish, and the fish wastewater did not show molecular signs of the SARS-CoV-2 virus, which indicates that in this “real-life scenario”, this environment does not seem to be an important means of propagation of the SARS-CoV-2 virus, which leaves transmission concentrated in the human-to-human environment and not using viral deposition on the surface of the fish, even though there was viral shedding and human infection, evidenced by the numbers of positive cases in the FVS-RCP database.

Although the fish did not show to be an intermediate reservoir of SARS-CoV-2, fish carry human cells, probably due to handling during the fishing and commercialization process. Xie and colleagues have demonstrated in their study that the SARS-CoV-2 virus supports the possibility of SARS-CoV-2 transmission through cold food handling, by verifying viral RNA copies in supernatant 72 hours post-infection [22]. Moreover, the amount of SARS-CoV-2 evidenced in aquatic organisms is low [23]. Among the viruses that have already been detected in fish, the White bream virus (WBV) [24, 25], Fathead Minnow Virus (FHMNV) [26, 27], and Chinook Salmon Bafinivirus (CSB) [23] stand out. All belong to the genus Banifivirus, which is named for their morphology. Coronaviruses have only been identified in aquatic mammals such as seals, dolphins, and beluga whales [23], evidence that was reinforced by the findings of Lam et al. [28], whose results showed that SARS-CoV-2 can infect a broad range of mammals, but not fish, birds, or reptiles, despite causing distress and affecting growth and development of neotropical fish when these were exposed to SARS-CoV-2 viral peptides [29].

It was not possible to detect the SARS-Cov-2 virus (Ct≤40, for Gene E) in the studied samples; however, an indicator of human contact was detected (Ct≤35, for the human RP Gene) in different types of analyzed materials (fish skin predominantly, air and fish wastewater from the open-air fish markets). Different types of human cells and secretions may carry the SARS-CoV-2 virus, the most predominant being the droplets originating from multi-ciliated cells in the upper respiratory tract. Nonetheless, it has been reported to be found in the stool, urine, and ocular secretions of infected individuals with viral load being important to viral shedding [30]. Moreover, the stability of the SARS-CoV-2 on the skin has been shown to vary (8h to 14 days) according to ambient temperature [31, 32]. These infected cells may be deposited on surfaces that have great contact with humans which indicates that the fish’s skin surface may serve as a potential source of deposited viral material.

In regions characterized by limited sewage and water treatment infrastructure, as observed in many low- and middle-income countries (LMICs), fecal and urinary shedding of viral particles pose a significant risk of contamination in open water sources. This issue is especially concerning near aquatic food markets, where contaminated water can facilitate zoonotic transmission and subsequent viral amplification within the surrounding ecosystem. The SARS-CoV-2 viral presence in wastewater, sewage, and other waterbodies has been shown in several studies [33–35]. It has been thought that environmental surveillance could be of use in epidemic and pandemic scenarios, with an early study in Japan showing that SARS-CoV-2 RNA was detected in a secondary-treated wastewater [36]. Studies investigating the presence of SARS-CoV-2 viral RNA in waters from the two major rivers, *Rio Negro* and *Rio Solimões*, which have different temperatures, pH, concentrations of organic material, etc., should be carried out to characterize viral shedding capabilities.

Cold-chain foods are also thought to be an important source of infection and have also become an issue in the COVID-19 pandemic [37]. Studies have found the presence of SARS-CoV-2 RNA in stored food from other countries. Chinese researchers evidenced the possible cross-border long-distance transmission of SARS-CoV-2, with the virus surviving on cold chain food and its packaging surface [38]. Feng and collaborators found that the higher the viral load, and the colder to environment used for food storage the more suitable it is for viral detection [10]. Fishery activities in Manaus provide fish to local markets, but also to long-distance consumer markets in Brazil and other countries. Despite this study not showing SARS-CoV-2 directly on the marketed fish *in natura* described here, cold-chain processing for long-distance exports could be a possible source of viable viral dissemination and should also be investigated.

According to Chen et al. [39], SARS-CoV-2 can infect a wide range of mammals, but there is still no evidence that it can infect aquatic organisms intended for human consumption. A major hypothesis arises from the phylogenetic comparison of the extracellular peptidase domain of the gene angiotensin I, converting enzyme 2 (ACE-2) which binds to the receptor binding domain (RBD) of the SARS-CoV-2 spike protein in a few species of fish. On the other hand, with the continuous emergence of new variants of the coronavirus, it is necessary to monitor the risks of aquatic animals becoming possible transmitters of this virus to humans.

The Amazonas state was one of the most impacted by COVID-19 in Brazil, with a health system collapse leading to high infection and mortality rates in mid-April 2020 [40] and in January 2021 [41]. According to Bolaño-Ortiz et al. [42], social inequality, low income, and poverty levels in cities like Manaus are directly related to the spread of SARS-CoV-2 in regional populations. The identification of possible host species of SARS-CoV-2 is essential to identify carriers of the new coronavirus, resulting in the need to increase surveillance in natural environments and prevent new outbreaks and future pandemics, as well as seeking to contribute to emergency measures to combat transmission of SARS-CoV-2 [39, 43] in locations where fish trading is an activity of diverse interest.

In the state of Amazonas and the North Brazilian region, large outdoor markets are common and of cultural importance, where the daily unloading of large quantities of fish for human consumption takes place. As it is an environment with high human circulation and a lot of fish handling, this study was designed to assess the possible contamination of fish by SARS-CoV-2, whether it comes from water, aerosols, or handling the fish by people infected with the virus in these open-air markets from the city of Manaus. In addition, it was possible to improve and standardize the extraction and molecular diagnostic kits, used in human samples, fish samples, aerosols, and wastewater, showing the efficiency and reliability of the analyses carried out in this project.

The perspectives of this study indicate that viral viability was not confirmed in a real-life scenario with the particularities of Manaus, highly humid with elevated temperatures. Future research should deepen the acquisition of material in different conditions and concentrations, as well as different strains of the virus, since the fact that animals, such as fish, serve as food and have a relevant commercial interest in the region, which leads them to be widely handled and circulated in the society.

## Supporting information

S1 Dataset Research database. Sample: sample type and fish species; Material: Materials used in sample collection; X: Positive RP gene in samples; Latitude and Longitude: specific geographic location of fishmarkets.
(XLSX)
